# Blended Versus Traditional Instruction for Pulpectomy Training: A Quasi-experimental, Multi-arm Study of Knowledge, Procedural Competence, and Satisfaction

**DOI:** 10.7759/cureus.98676

**Published:** 2025-12-08

**Authors:** KC Vignesh, Vivek K, Selvakumar Haridoss, Kavitha Swaminathan, Srivasunthra Srinivasan, Sanjana Sree Manisekar, Anupriyadharshini S

**Affiliations:** 1 Pediatric and Preventive Dentistry, Sri Ramachandra Dental College and Hospital, Sri Ramachandra Institute of Higher Education and Research (Deemed to be University), Chennai, IND; 2 Dentistry, Sri Ramachandra Dental College and Hospital, Sri Ramachandra Institute of Higher Education and Research (Deemed to be University), Chennai, IND

**Keywords:** flipped classroom, online learning, osce, pediatric endodontics, problem-based learning, pulpectomy, quasi-experimental, sdg 4: quality education, undergraduate dental education

## Abstract

Background

Blended instructional designs (flipped classroom, problem-based learning (PBL), and online-guided learning) are widely adopted in health-professions education, but evidence on their impact on practical skills in pediatric endodontics remains limited.

Objective

The main objective of this study is to compare knowledge, procedural skill (objective structured clinical examination (OSCE)), and student satisfaction across four pedagogies for undergraduate pulpectomy training, while holding contact time, hands-on exposure, and faculty-to-student ratios constant.

Methods

A quasi-experimental, parallel-group study at Sri Ramachandra Institute of Higher Education and Research (SRIHER), Chennai, India (January-October 2025). Interns were taught pulpectomy using one of four arms: traditional (control), glipped, PBL, or online-guided (SRIHER learning management system (LMS)). Outcomes were (i) knowledge (pre-/post-test MCQ test; prespecified analysis using analysis of covariance (ANCOVA), with post-test as the outcome, arm as the factor, and pre-test as the covariate), (ii) OSCE (weighted 50-point checklist converted to percentage; competency ≥60%), and (iii) satisfaction (Likert 1-5; subscales: engagement, clarity, confidence, applicability, and overall).

Results

Eighty students were included (n = 20 per arm). OSCE performance was high and similar across arms, with overlapping 95% CIs: traditional 69.0 ± 3.5%, flipped 68.2 ± 3.5%, PBL 69.5 ± 3.8%, and online-guided 68.5 ± 3.8%; overall 68.8 ± 3.6%. Competency (≥60%) was 100% in every arm (20/20 each). Satisfaction was uniformly high: overall (O1) was 5/5 across all students, and subscales were in the “agree-strongly agree” range. Knowledge scores showed modest pre-post improvement across arms (overall mean gain +0.43 points on a five-point scale), and change scores did not differ significantly between teaching methods (one-way analysis of variance (ANOVA) on gain scores, p = 0.515).

Conclusions

When instructional “dose” and hands-on practice are standardized, traditional, flipped, PBL, and online-guided formats yield comparable, high procedural competence and a uniformly positive learner experience for pulpectomy training. Curricular structure ensuring adequate guided practice may be more consequential for immediate skill than the specific didactic format. Multi-center randomized studies with standard settings and longer-term outcomes (retention and clinical transfer) are warranted.

## Introduction

Undergraduate dental curricula must simultaneously build theoretical understanding and psychomotor competence, yet large cohorts, limited contact time, and the complexity of clinical skill transfer often constrain traditional lecture-based delivery [1]. Blended learning - typically combining pre-class online materials with in-class active, case-based engagement (e.g., flipped classroom) - has been proposed to make learning more student-centered and to better support clinical reasoning and skills integration in dentistry [2].

In a conservative dentistry course comparing consecutive cohorts, a flipped/blended model yielded significantly higher performance across written and online exams, quizzes, assignments, and clinical assessments, with students also reporting high satisfaction - evidence that structured blending can enhance outcomes in clinical dental education [3]. Comparable educational trials in clinical skills (for example, peer versus professional training in basic life support) illustrate how randomized or quasi-experimental designs can clarify modality effects on competence and learner outcomes [4]. Complementing this, a systematic review focused on orthodontics and pediatric dentistry concluded that e-learning is generally as effective as traditional methods and well accepted by learners, supporting a blended approach as an effective complement to face-to-face teaching [5]. Prior work validating digital learning and assessment tools further supports integrating learning management system (LMS)-based modules into procedural curricula and reporting their metrics transparently [6]. Notably, individual studies within that review show mixed short-term knowledge differences between flipped and conventional formats while maintaining strong learner preference for blended/flipped delivery - underscoring that design details and context matter [7].

Despite encouraging evidence, prior syntheses highlight heterogeneity in populations, interventions, and outcome measures that have limited quantitative pooling, and there remains a need for discipline-specific, skill-focused evaluations using standardized knowledge and performance metrics in undergraduate dentistry [8]. Within procedural domains such as pediatric endodontics, rigorous pre-/post-knowledge testing is common and feasible for capturing short-term learning gains, supporting its inclusion alongside practical performance assessments and learner feedback in blended-learning evaluations. Accordingly, this quasi-experimental, multi-arm study compared traditional lecture-based teaching, flipped classroom, problem-based learning (PBL), and online-guided formats for undergraduate pulpectomy training while holding contact time, hands-on exposure, and faculty-to-student ratios constant. Our primary hypothesis was that, under equivalent hands-on conditions, the three blended formats would be non-inferior to traditional teaching for immediate pulpectomy objective structured clinical examination (OSCE) performance, with broadly similar knowledge gains and student satisfaction.

## Materials and methods

Study design and ethical approval

This quasi-experimental, parallel-groups study was conducted in the Department of Pediatric and Preventive Dentistry, Sri Ramachandra Institute of Higher Education and Research (SRIHER), Chennai, India, from January to October 2025. Four instructional arms delivered the same pulpectomy curriculum: traditional (control), flipped classroom, PBL, and online-guided. Interns were allocated to these arms using a quasi-experimental approach based on existing timetable blocks rather than individual randomization, and group allocation was therefore not concealed.

The study was approved by the Institutional Ethics Committee (IEC) of SRIHER (approval no. CSP-III/25/JUN/23/285) and conducted in accordance with institutional guidelines and the Declaration of Helsinki. Written informed consent was obtained from all participants, and no personally identifiable information is included.

Participants

**Figure 1:**
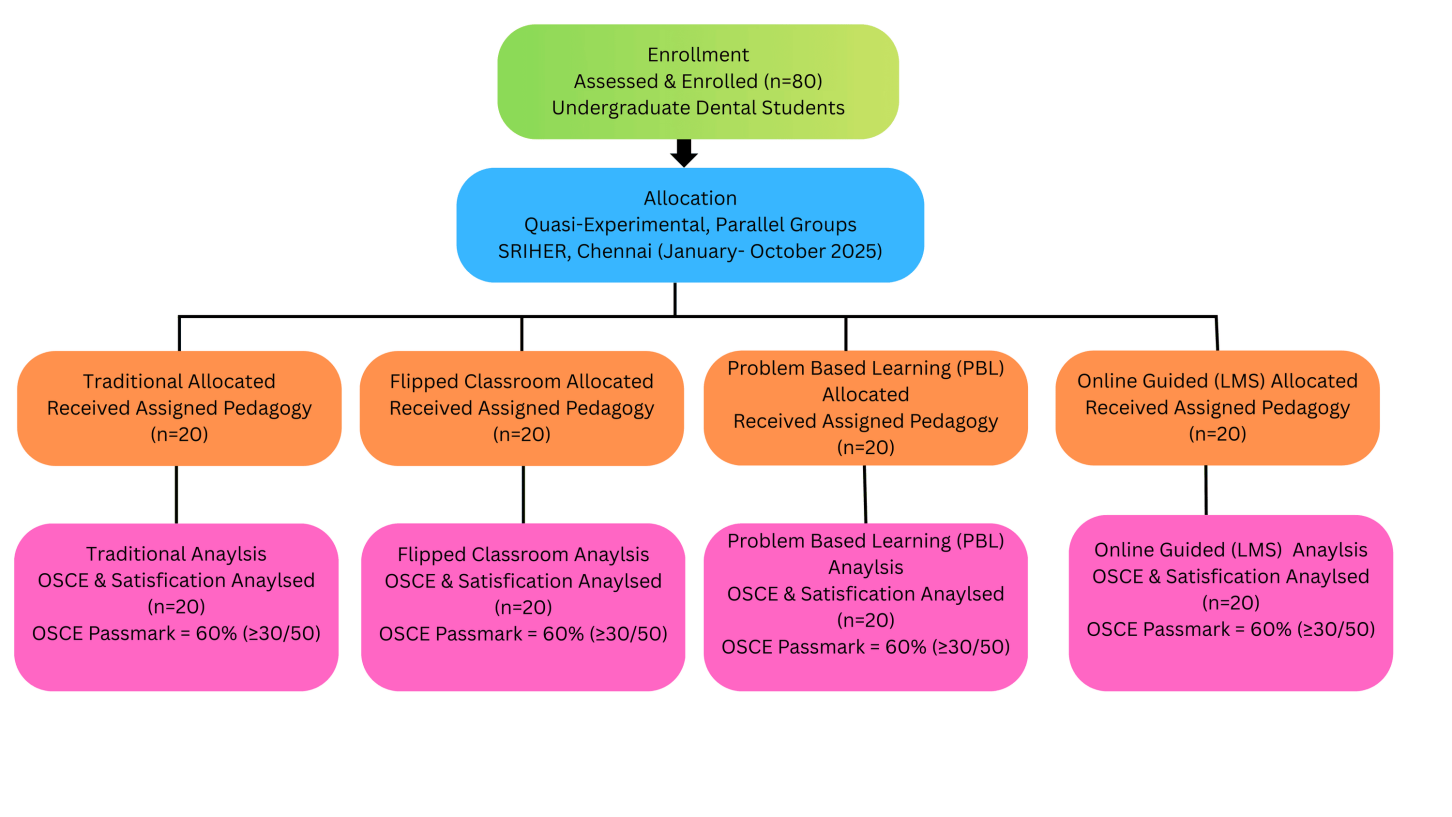
Participant flow through a four-arm quasi-experimental pulpectomy training study Enrollment (N = 80) → allocation to traditional, flipped classroom, PBL, and online-guided arms (n = 20 each), with identical contact time, hands-on exposure, and faculty-to-student ratios. OSCE scored out of 50 and converted to a percentage; competency ≥60%. Satisfaction was measured on five-point Likert scales. PBL, problem-based learning; LMS, learning management system; OSCE, objective structured clinical examination

Undergraduate dental interns scheduled for the pulpectomy teaching block were invited. Inclusion criteria were enrollment and consent, while exclusion criteria were prior advanced or extramural pulpectomy training or inability to attend teaching and assessments; the sample size was 80 interns (20 per arm), determined a priori by cohort capacity to ensure balanced groups. Participant enrollment, allocation to the four pedagogic arms (traditional, flipped classroom, PBL, and online-guided; n = 20 each), and numbers analyzed are summarized in Figure 1.

Interventions

Students received pulpectomy instruction via one of four pedagogic arms. The traditional arm comprised in-person didactic lecture(s), followed by supervised hands-on practice. The flipped classroom arm provided pre-class micro-lectures/readings, released ≥48 hours in advance, with in-class application activities and the same hands-on component. The PBL arm used facilitated small-group case triggers mapped to the learning objectives, with the same hands-on component. The online-guided arm delivered interactive modules on the SRIHER e-learning LMS (https://hselearning.sriher.com/login/index.php) with embedded knowledge checks; hands-on activities were identical. The LMS recorded module access and completion status; however, fine-grained time-on-task analytics were not consistently archived and are therefore not reported. Across all four arms, the total scheduled contact time for pulpectomy teaching was held constant at two hours, comprising one hour of preparatory activities and one hour of supervised hands-on practice. The same simulation facility, instruments, and training teeth were used in all groups, and faculty-to-student ratios were maintained at approximately 1:5 to minimize variability in supervision and feedback.

Outcomes and measurements

The primary outcome was knowledge, assessed with a blueprint-matched five-item MCQ test administered pre- and post-instruction (see Appendix 1); items targeted core pulpectomy learning objectives (indications and case selection, access cavity design, working length determination, canal preparation and irrigation, and obturation principles), and total scores were expressed as the number of correct responses (0-5), with a change score (Δ = Post - Pre) derived. A brief five-item format was chosen to fit within the time constraints of the teaching session while focusing on the most critical cognitive steps in pulpectomy. Internal consistency (Cronbach’s α) was planned. Secondary outcomes were (i) procedural skill, measured using a weighted pulpectomy OSCE checklist (0-5 per item; total converted to a percentage of 50 points), with competency defined a priori as OSCE ≥60% (≥30/50); the ≥60% threshold corresponded to the institutional pass standard for undergraduate clinical examinations and was adopted as the minimum competence benchmark for this study. OSCE performance was rated by a single experienced pediatric dentistry faculty member; formal inter-rater reliability could not be estimated, and (ii) student satisfaction, captured on five-point Likert items for engagement (E1-E3), clarity (C1-C3), confidence (S1-S3), applicability (A1-A2), and overall (O1). Satisfaction items were summarized descriptively as counts, percentages, and key distributions by arm; internal consistency (Cronbach’s α) was planned [9].

Data management

Knowledge and satisfaction data were collected using institutional forms, and OSCE ratings were recorded on standardized checklists. All data were exported to CSV, range-checked for quality, and stored on a secure institutional drive with restricted access.

Statistical analysis

Analyses were performed in Stata SE 18.0 (StataCorp LLC, College Station, TX, USA). The prespecified primary model for knowledge was analysis of covariance (ANCOVA), with post-test score as the dependent variable, arm as the fixed factor, and pre-test score as the covariate; adjusted mean differences with 95% CIs and partial η² were planned. Sensitivity analyses included Δ-ANOVA (analysis of variance) on change scores, where normality or variance assumptions were not met (Shapiro-Wilk and Levene), and Kruskal-Wallis with Dunn’s post-hoc (Bonferroni) was planned. Complete paired knowledge data (pre- and post-test) were available for 80 interns (n = 20 per arm). We analyzed knowledge gain (Δ = Post - Pre) using one-way ANOVA with teaching method as the factor, preceded by Shapiro-Wilk normality checks. OSCE percentages were compared across arms using one-way ANOVA (or Kruskal-Wallis when appropriate), and competency proportions were summarized with 95% CIs. Satisfaction items were summarized descriptively, and internal consistency was assessed using Cronbach’s α. Two-sided α = 0.05 was used throughout. The study was non-randomized, and no imputation was planned.

## Results

A total of 80 undergraduate dental interns were included (n = 20 per arm: traditional, flipped classroom, PBL, and online-guided).

Primary outcome

Knowledge

Complete paired knowledge data (both pre- and post-test) were available for 80 interns (n = 20 per arm). Across arms, pre-test scores ranged from 2.71 to 3.57, and post-test scores from 3.43 to 3.71 (Table 1).

**Table 1:** Descriptive statistics of pre-test, post-test, and knowledge-gain scores by teaching method

Teaching method	n	Pre-test mean ± SD	Post-test mean ± SD	Mean gain (Post-Pre) ± SD
Traditional	20	2.71 ± 1.11	3.57 ± 0.79	0.86 ± 1.21
Flipped classroom	20	3.29 ± 1.11	3.71 ± 0.49	0.43 ± 1.13
Problem-based learning (PBL)	20	3.00 ± 0.82	3.43 ± 0.79	0.43 ± 0.53
Online-guided	20	3.57 ± 0.79	3.57 ± 0.79	0.00 ± 1.15
Overall	80	3.14 ± 0.97	3.57 ± 0.69	0.43 ± 1.03

Overall, knowledge increased from 3.14 ± 0.97 at pre-test to 3.57 ± 0.69 at post-test, corresponding to a mean gain of +0.43 ± 1.03 points on the five-point scale. Gain scores approximated normal distributions within each teaching method (Shapiro-Wilk p = 0.06-0.99). One-way ANOVA on gain scores showed no statistically significant differences between teaching methods (F(3,76) = 0.78, p = 0.515). Tukey HSD post-hoc tests confirmed that all pairwise differences in mean gain had 95% CIs crossing zero, indicating similar short-term knowledge gains across traditional, flipped, PBL, and online-guided formats.

Secondary outcome

Procedural Skill (OSCE)

OSCE performance (converted to percentage) was high and similar across arms, with overlapping 95% CIs: traditional 69.0 ± 3.5 (95% CI: 67.4-70.6), flipped 68.2 ± 3.5 (95% CI: 66.6-69.8), PBL 69.5 ± 3.8 (95% CI: 67.7-71.3), online-guided 68.5 ± 3.8 (95% CI: 66.7-70.3); overall 68.8 ± 3.6 (95% CI: 68.0-69.6). Using the pre-specified pass mark (≥60%), competency was 100% in every arm (20/20 each; 80/80 overall) (Table 2).

**Table 2:** OSCE outcomes by arm OSCE scored out of 50 and converted to percentage: percentage = (total/50) × 100. Pass mark = 60% (≥30/50). Competency is calculated as the percentage of students with OSCE ≥60%. 95% CIs are two-sided, computed as mean ± t × (SD/√n), with df = 19 for each arm (t ≈ 2.093) and df = 79 for overall (t ≈ 1.990). Single assessor; inter-rater reliability not estimated. OSCE, objective structured clinical examination

Arm	N	OSCE total (%) mean	SD	95% CI (lower)	95% CI (upper)	Competent n	Competent %
Traditional	20	69.0	3.5	67.4	70.6	20	100.0
Flipped classroom	20	68.2	3.5	66.6	69.8	20	100.0
Problem-based learning (PBL)	20	69.5	3.8	67.7	71.3	20	100.0
Online-guided	20	68.5	3.8	66.7	70.3	20	100.0
Overall	80	68.8	3.6	68.0	69.6	80	100.0

Student Satisfaction

Satisfaction ratings were uniformly high across arms. The distribution of key items - confidence (S1), applicability of online resources (A1), and overall satisfaction (O1) - by teaching method is shown in Table 3; almost all students selected “agree” or “strongly agree” for S1 and A1, and O1 was 5/5 for every participant.

**Table 3:** Distribution of key satisfaction items by arm Values are n (%). S1 = confidence item; A1 = applicability of online resources; O1 = overall satisfaction. Likert anchors range from 1 (strongly disagree) to 5 (strongly agree).

Arm	N	S1 (confidence) = 5, n (%)	S1 = 4, n (%)	A1 (online resources helpful) - filled?	A1 = 5, n (%)	A1 = 4, n (%)	O1 (overall satisfaction = 5), n (%)
Traditional	20	2 (10.0)	18 (90.0)	No (not used)	-	-	20 (100.0)
Flipped classroom	20	3 (15.0)	17 (85.0)	Yes	0 (0.0)	20 (100.0)	20 (100.0)
Problem-based learning (PBL)	20	0 (0.0)	20 (100.0)	No (not used)	-	-	20 (100.0)
Online-guided	20	1 (5.0)	19 (95.0)	Yes	1 (5.0)	19 (95.0)	20 (100.0)
Overall	80	6 (7.5)	74 (92.5)	-	-	-	80 (100.0)

Applicability items were completed only for arms with online components (flipped and online-guided).

## Discussion

We selected a quasi-experimental, parallel-group design to compare four established instructional approaches (traditional, flipped, PBL, and online-guided) under identical contact time, hands-on exposure, and faculty-to-student ratios, thereby isolating pedagogy rather than dose. Prior dental-education studies show that blending asynchronous preparation with interactive sessions can heighten engagement and assessment performance compared with lecture-based learning, supporting our inclusion of an online-guided arm delivered through an LMS alongside a traditional control [10]. Case-based and problem-based formats have repeatedly matched or exceeded lectures on written knowledge and learner satisfaction in dental curricula, justifying a PBL arm [11]. Finally, internet-based or online-supported teaching has demonstrated equivalence to face-to-face seminars for targeted cognitive and practical objectives, validating the use of LMS-hosted pre-class materials and our OSCE as a competency endpoint; adjusting post-test for baseline via ANCOVA is standard in education trials to reduce bias from initial differences [12].

Our pattern of similar OSCE performance across arms, with uniformly high satisfaction, aligns with several recent dental-education reports showing that flipped, PBL, and online-supported models produce comparable or modestly improved procedural/skill outcomes relative to lectures when implementation fidelity and contact time are matched [11]. Multiple studies also report consistently high student satisfaction with blended/flipped formats, often citing flexibility and perceived relevance, mirroring our ceiling-level O1 ratings [13-15]. Conversely, some evaluations have found no meaningful between-arm differences in summative performance despite positive perceptions, particularly when curricula are tightly standardized or when ceiling effects limit discriminability, again consistent with our OSCE results [11,16-18]. This heterogeneity across studies likely reflects differences in course design, assessment blueprinting (knowledge vs skills), and learner readiness.

Three signals stand out. First, procedural competence (OSCE%) was high and statistically indistinguishable across all four pedagogies, indicating that when hands-on time and resources are held constant, how we prime students (lecture vs flipped vs PBL vs online-guided) may matter less for immediate technical performance. Second, overall satisfaction was at ceiling (5/5 for all), which is reassuring but also limits sensitivity to detect differences - a classic ceiling effect. Third, knowledge scores showed modest pre-post improvement across arms, with no significant differences in gain between teaching methods, aligning with literature indicating that preparatory activities and structured in-class application can lift short-term cognitive outcomes even when different delivery formats are used [10,19-21].

Strengths include a multi-arm, parallel design; strict control of contact time, hands-on exposure, and resources across pedagogies; and triangulation of outcomes across knowledge, procedural skill (OSCE), and satisfaction. However, several limitations must be acknowledged. First, the non-randomized, quasi-experimental allocation of students to arms means that residual confounding and allocation bias cannot be excluded. Second, OSCE ratings were provided by a single, non-blinded assessor, and inter-rater reliability could not be estimated; both factors may introduce measurement bias in procedural scores. Third, although the MCQ test was blueprint-matched to key pulpectomy concepts, its brief five-item format necessarily limits psychometric sensitivity to detect nuanced cognitive differences between modalities. Fourth, satisfaction scores and OSCE competence showed marked ceiling effects (100% ≥ 60%), which is consistent with reports of ceiling effects in patient- and learner-reported outcome measures and underlines the need for validated, sensitive patient-reported outcome measures (PROMs) in educational research [22]. Finally, this was a single-site study with immediate post-instruction outcomes only, limiting generalizability and precluding conclusions about long-term retention, clinical transfer, or cost-effectiveness.

Implications and future directions

Future work should prioritize multi-center randomized or stepped-wedge designs; incorporate standard-setting methods (e.g., Modified Angoff/Borderline Regression) above the fixed 60% cut-off; and extend follow-up to retention and transfer (clinic performance and patient outcomes). Importantly, apparent equivalence in immediate technical performance across pedagogies does not imply equivalence in long-term knowledge retention, clinical behavior change, or economic outcomes. Learning analytics from the LMS (time-on-task and mastery pathways) could be leveraged to individualize preparation and to test dose-response relationships. Finally, cost-effectiveness, faculty workload, and equity of access (bandwidth and device constraints) warrant explicit evaluation in low- and middle-income countries (LMICs) settings highlighted in recent reports [11,20,21].

## Conclusions

Across four pedagogy arms with matched hands-on exposure, interns achieved equivalent, high OSCE performance and uniformly high satisfaction. These findings relate specifically to immediate post-instruction performance in a single institution and should not be interpreted as demonstrating long-term equivalence of the four pedagogies for retention, clinical transfer, or cost-effectiveness. Modest within-arm knowledge gains were observed, without significant differences in gain between teaching methods. Future multi-center, randomized work with robust standard setting and longer-term outcomes is needed to confirm these findings and define when blended or PBL approaches provide measurable advantages over traditional delivery.

## Appendices

Appendix 1. Blueprint-matched multiple-choice knowledge test on pulpectomy

Item 1. Indication of pulpectomy EXCEPT: A. Continuous pain B. Controlled haemorrhage from amputated coronal pulp C. Sinus tract D. Suppuration canals

Item 2. Access opening in primary teeth is dependent on these factors EXCEPT: A. Size of the pulp chamber B. Shape of the pulp chamber C. Number, position and curvature of the root canals D. Age of the patient

Item 3. Sodium hypochlorite irrigation as per AAPD recommendations: A. 0.2-2% B. 2% C. 1% D. 1.5%

Item 4. Canal enlargement in primary teeth with stainless steel file can be done till: A. 25-30 size B. 45-50 size C. 50-60 size D. 60-70 size

Item 5. AAPD recommendation for isolation: A. Cotton rolls B. Saliva ejectors C. Rubber dam D. Absorbing papers
